# Microcapsule-Type Self-Healing Protective Coating for Cementitious Composites with Secondary Crack Preventing Ability

**DOI:** 10.3390/ma10020114

**Published:** 2017-01-26

**Authors:** Dong-Min Kim, Hwan-Chul Yu, Hye-In Yang, Yu-Jin Cho, Kwang-Myong Lee, Chan-Moon Chung

**Affiliations:** 1 Department of Chemistry, Yonsei University, Wonju, Gangwon-do 26493, Korea; dmkimr@yonsei.ac.kr (D.-M.K.); taken4@nate.com (H.-C.Y.); monkey0627@hanmail.net (H.-I.Y.); chyujn904@naver.com (Y.-J.C.); 2 Department of Civil and Environmental Engineering, Sungkyunkwan University, Suwon, Gyeonggi-do 16419, Korea; leekm79@skku.edu

**Keywords:** microcapsule, self-healing protective coating, secondary crack prevention, cementitious materials

## Abstract

A microcapsule-type self-healing protective coating with secondary crack preventing capability has been developed using a silanol-terminated polydimethylsiloxane (STP)/dibutyltin dilaurate (DD) healing agent. STP undergoes condensation reaction in the presence of DD to give a viscoelastic substance. STP- and DD-containing microcapsules were prepared by in-situ polymerization and interfacial polymerization methods, respectively. The microcapsules were characterized by Fourier-transform infrared (FT-IR) spectroscopy, optical microscopy, and scanning electron microscopy (SEM). The microcapsules were integrated into commercial enamel paint or epoxy coating formulations, which were applied on silicon wafers, steel panels, and mortar specimens to make dual-capsule self-healing protective coatings. When the STP/DD-based coating was scratched, self-healing of the damaged region occurred, which was demonstrated by SEM, electrochemical test, and water permeability test. It was also confirmed that secondary crack did not occur in the healed region upon application of vigorous vibration to the self-healing coating.

## 1. Introduction

A protective coating is utilized to protect a material from various deterioration factors. A typical example of this is protective coatings for cementitious composites such as concrete and mortar that provide barrier protection against the intrusion of water, chloride ion, carbon dioxide, and other corrosive substances. However, if the protective coating is damaged by microcracking or scratching, those substances would penetrate through damaged holes. This causes the deterioration of the cementitious materials, leading to significant reduction of their serviceability. If the protective coating can automatically heal the damaged region, it would very effectively protect the materials from deterioration. Self-healing technology would provide improved public safety and reduced maintenance cost [1,2,3,4,5]. Although many studies of self-healing anticorrosive coatings for metal protection have appeared [6,7,8,9], reports on self-healing coatings for cementitious composites are very rare [10,11].

Song et al. previously reported a microcapsule-type protective coating system for concrete application having a sunlight-induced self-healing capability [10]. When the coating system is damaged, a healing agent is released from ruptured microcapsules and fills the damaged region. The healing agent generates a hard solid through photo-cross-linking, resulting in the recovery of the sealing function. However, the coating system has a limitation, in that it cannot be healed without UV light irradiation. A new concrete coating system that can be self-healed in a dark place is needed for wider application. Another limitation of the previous system is that the healing agent turns into a hard solid through the healing reaction. When microcracking occurs again in the healed region, the self-healing cannot be repeated—not only because the microcapsules are depleted, but also because the solidified healing agent is a hard solid with no additional self-healing ability.

Self-healing protective coatings on bridges, tunnels, and buildings can be exposed to intense vibration induced by heavy traffic. For example, it has been shown that civil engineering structures such as bridges are usually exposed to traffic-induced vibration of 2–10 Hz [12]. The vibrations can cause damage such as microcracking and crazing. After the first self-healing event, secondary damage may occur in the healed region under continuous vibration, probably inside the solidified healing agent or in boundary between the solidified healing agent and coating matrix. Yang et al. reported a self-healing anticorrosive coating for metal application having secondary damage-preventing capability utilizing a healing agent that turns into a viscoelastic organogel material [13]. It is known that vibration energy effectively dissipates in a viscoelastic material because of its viscosity and elasticity, leading to the maintenance of its shape [14].

In this paper, a new microcapsule-type self-healing protective coating for concrete application has been developed: the coating system can be self-healed without UV irradiation, and can prevent secondary cracking. A silanol-terminated polydimethylsiloxane (STP)/dibutyltin dilaurate (DD) combination was used as a healing agent, because the two components react to give a viscoelastic substance. The new STP/DD-based self-healing system can offer an additional advantage of minimized loss of healing agent during the healing event, while the self-healing of the previous organogel-based system occurs by evaporation of a relatively large amount of solvent [13]. STP and DD were separately microencapsulated, and the resulting microcapsules were embedded in a coating matrix to prepare a dual-capsule self-healing coating. The self-healing and secondary damage-preventing ability of the new coating system was demonstrated, and its preliminary evaluation was carried out for mortar specimens.

## 2. Materials and Methods

### 2.1. Materials

Urea, formaldehyde solution (37 wt %), poly(ethylene-*alt*-maleic anhydride) (EMA), resorcinol, 1-octanol, ammonium chloride, tolylene 2, 4-diisocyanate (TDI), 1,4-butanediol (BD), ethylene glycol (EG), gum arabic, cyclohexanone, and chlorobenzene were used in microencapsulation process. Silanol-terminated polydimethylsiloxane (STP) (DMS-S12) and dibutyltin dilaurate (DD) were used as core materials. Gum arabic dispersed in de-ionized water was used as surfactant. An enamel paint formulation (KCI 720, white) and an epoxy coating formulation (KU-420K40) were used as coating materials. All solvents were purified using calcium sulfate as dry agent. Mortar specimens were prepared with a mass ratio of cement:sand:water = 2:6:1 according to a KSF2476 standard method. Mortar paste was first cured in a mold for 48 h at room temperature. Each mortar was further cured for 5 days in water, and then finally cured for 7 days under ambient conditions.

### 2.2. Instruments

Gel permeation chromatography (GPC) analysis was carried out using a doubly-connected Showa Denko Shodex KF-806L column at 100 °C and an eluent of *N*-methyl-2-pyrrolidone (NMP) at a flow rate of 1.0 mL/min; the results were calibrated with respect to polystyrene standards. A mechanical stirrer (NZ-1000, Eyela, Tokyo, Japan) was used in microencapsulation. A microscope (BX-51, Olympus, Tokyo, Japan) was used to take pictures of the microcapsules. The microcapsule size was analyzed using a CCD camera (CC-12, Olympus, Tokyo, Japan) equipped in the microscope. Image analysis software (analySIS TS, Olympus, Tokyo, Japan) IR spectra were recorded on a Fourier transform infrared (FT-IR) spectrophotometer (Spectrum One B, Perkin Elmer Co., Waltham, MA, USA). A field emission scanning electron microscope (FE-SEM) (SU-70, Hitachi, Quanta FEG 250, FEI, Tokyo, Japan) was used to examine the morphology of the microcapsules and coating surface. A potentiostat/galvanostat (263A, Ametek, Berwyn, PA, USA) was used to examine the conductivity of the coated substrate. A biaxial simultaneous vibration tester (BF-50UD, IDEX, Lake Forest, IL, USA) was used to apply vibration to coating specimens. An advanced Rheometric Expansion System (ARES, Rheometric Scientific, Piscataway, NJ, USA) was used to examine the viscoelasticity of the reacted STP. Thermal analysis was carried out on a Shimadzu TGA-50 (Shimadzu Corp., Kyoto, Japan) at a heating rate of 10 °C/min under nitrogen.

### 2.3. Synthesis of Prepolymer

TDI (11.497 g, 0.066 mol) was dissolved in cyclohexanone (70 mL) under nitrogen atmosphere in a 150-mL round-bottom flask. The flask was suspended and heated in an oil bath, and BD (2.704 g, 0.030 mol) was slowly added at 55 °C. The resultant mixture was allowed to react at 80 °C for 24 h. The reaction mixture was evaporated at 80 °C for 3–4 h and then at 100 °C for 1 h in a vacuum oven to remove cyclohexanone, water, and excess TDI. A prepolymer was obtained as a viscous yellow oil.

### 2.4. Microencapsulation of DD

A gum arabic (3.10 g) surfactant was dissolved in deionized water (20 mL) in a 100-mL beaker to obtain Solution A. On the other hand, the prepolymer (3.00 g) was dissolved in chlorobenzene (32.00 g) and DD (1.00 g) was then added. The resultant mixture was slowly poured into Solution A to give an emulsion, which was then heated. Ethylene glycol (3.00 g) as a chain extender was slowly added to the emulsion at 50 °C. When the temperature reached 70 °C, the reaction was stopped. After filtration, the microcapsules were rinsed with de-ionized water and air-dried for 72 h (yield = 92%).

### 2.5. Microencapsulation of STP and Methacryloxypropyl-Terminated Polydimethylsiloxane (MTP)

In a 100-mL beaker, water (20 mL) and a 2.5 wt % aqueous solution of EMA (5 mL) were added. The beaker was placed in a temperature-controlled water bath equipped with a mechanical stirrer. Under agitation at 300 rpm, urea (0.503 g), ammonium chloride (0.050 g), and resorcinol (0.050 g) were added. The pH of the resulting mixture was adjusted to 3.5 using a 10% NaOH solution. The mixture was agitated at 1000 rpm, and two drops of 1-octanol were added. STP (8 mL) was added, and the resultant mixture was maintained for 15 min to form a stable emulsion. After adding 37% formaldehyde (1.456 g) solution, the temperature of the mixture was increased to 55 °C and maintained for 4.5 h. The reaction mixture was cooled to room temperature, and the microcapsules were isolated by vacuum filtration. The microcapsules were washed with water and THF, and air-dried for 48 h (yield = 87%). MTP-loaded microcapsules were prepared according to a previously reported method [10].

### 2.6. Measurement of Microcapsule Core/Shell Ratio

After weighing, the STP-containing microcapsules were crushed by pressing them in a 5-mL vial and washed with ethanol twice. The ruptured shell material was dried under ambient conditions and weighed. In the case of DD-loaded microcapsules, additional thermogravimetric analysis (TGA) study was performed to investigate the mass ratio of chlorobenzene/DD.

### 2.7. Viscoelasticity Test

The STP and DD were mixed with a mass ratio of 10:1, and the mixture was poured in molds with 25-mm diameter and 1-mm height and allowed to react for 4 weeks. Disk-like specimens of the reacted STP were separated from the molds. The viscoelasticity of the reacted STP was examined using ARES. This experiment was conducted under conditions where strain and temperature were held constant, while frequency was varied from 0.1 to 510.0 rad/s. Then, experimental results were plotted by G’ and G” versus frequency.

### 2.8. Preparation of the Coating Samples

The STP- and DD-containing microcapsules were mixed into the commercial enamel paint (or epoxy coating) formulation with a mass ratio of STP capsules:DD capsules:paint of 19:6:75. The resulting formulations were applied to the surface of the silicon wafer, steel panel, or mortar specimen, and then dried for 3–4 days at room temperature. Control coating samples were prepared in a similar fashion without microcapsules. MTP-based self-healing coatings were prepared by mixing the MTP-loaded microcapsules and the commercial enamel paint (or epoxy coating) formulation with a mass ratio of 1:4. A scratch was generated in the coated surface with a razor blade. The scratched STP/DD-based samples were allowed to heal for 4 h. The scratched MTP-based coatings were photoirradiated with a xenon lamp for 1.5 h.

### 2.9. Application of Vibration

The coated silicon wafers and steel plates were attached on the top surface of the vibration tester. Vibration was applied in transportation mode with a frequency range of 10–30 Hz. Sweep time and cycle were 15 min and 4 cycles, respectively.

### 2.10. Electrochemical Test

Self-healing coatings and control coatings were prepared on steel panels. Electrochemical tests were conducted by using a computer-controlled potentiostat/galvanostat (model 263A, Advanced Measurement Technology) in a conventional three-electrode electrochemical cell equipped with a platinum counter electrode, an Ag/AgCl electrode in a saturated NaCl aqueous solution as the reference electrode, and the coated steel panel as the working electrode (Figure S1). The steady-state conduction between the coated metal substrate and a platinum electrode held at 3 V through an aqueous electrolyte (1 mol/L NaCl) was measured. The current passing through the specimen was recorded by using Electrochemistry PowerSuite software (Advanced Measurement Technology, Oak Ridge, TN, USA). For each kind of sample, at least five specimens were used, and an average of the measured values was taken.

### 2.11. Water Permeability Test

The self-healing and control coatings were applied to one rectangular side of 40 mm × 40 mm × 130 mm square column mortars. Four side surfaces adjacent to the coated side were covered with epoxy resin to prevent water permeation through the proximate surfaces, and the scratched surface was brought into contact with water. After 48 h, the increase in mass due to water absorption was determined. Three specimens were tested for each kind of sample, and an average of the measured values was taken.

## 3. Results and Discussion

### 3.1. Healing Agent

**Scheme 1:**
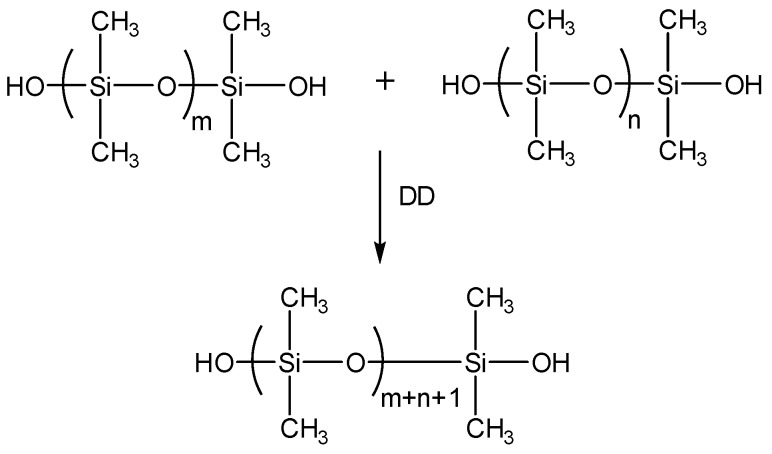
Condensation reaction of silanol-terminated polydimethylsiloxane (STP) in the presence of dibutyltin dilaurate (DD).

**Figure 1:**
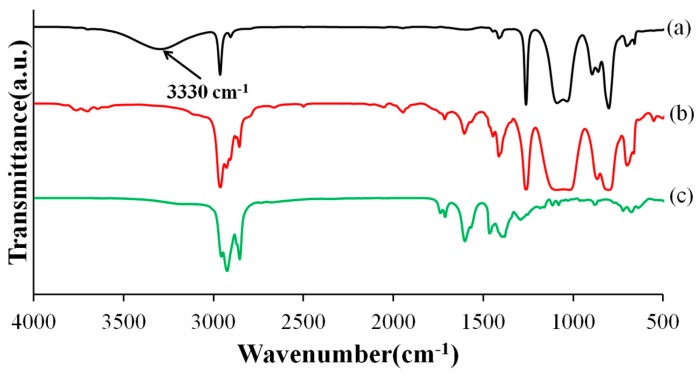
Infrared spectra of (**a**) a pristine STP; (**b**) a reaction product of STP/DD 10:1 mixture; and (**c**) DD.

In this study, an STP/DD combination was used as a healing agent. STP is a polydimethylsiloxane having silanol end groups. A polydimethylsiloxane structure was chosen because it is hydrophobic and water repellent. STP was expected to undergo condensation reaction of silanol groups in the presence of DD to give a higher molecular weight polydimethylsiloxane (Scheme 1) [15]. In IR spectra of a pristine STP and a reaction product (Figure 1), an absorption band at 3330 cm^−1^ due to silanol groups disappeared after the reaction of STP in the presence of DD, indicating that the condensation reaction of STP proceeded. The pristine STP is a low-viscosity liquid and turned into highly viscous or viscoelastic substances through the reaction.

To determine the mass ratio of STP to DD suitable for self-healing, mixtures with different ratios of the two compounds were reacted. Each mixture of 1–1.5 mL volume was allowed to react for 4 weeks at room temperature. STP/DD 2:1 and 5:1 mass ratio mixtures underwent phase separation, and 20:1 and 50:1 mixtures afforded viscous liquids. Viscoelastic substances were generated from STP/DD mixtures with a mass ratio range 8:1–12:1, so this ratio range was selected for the preparation of the self-healing system. It was found that the reaction time of the mixture greatly depends on its quantity. When a small amount (approximately 0.01 mL) of STP/DD 10:1 mixture was tested, it took only 4 h for the mixture to fully react to give a viscoelastic substance. It was postulated that 4 h is enough time to self-heal, because a very small amount of STP and DD would be released from ruptured microcapsules in the damaged region.

**Figure 2:**
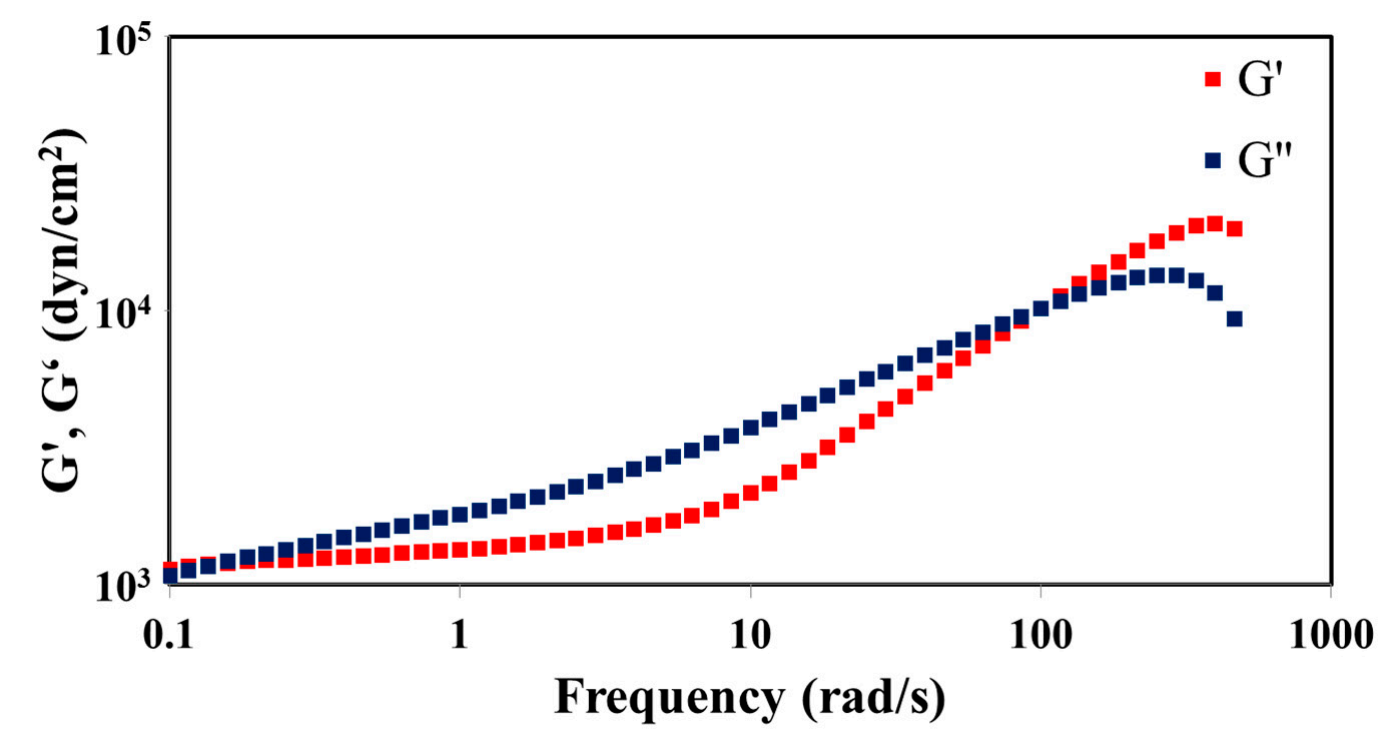
Storage modulus (G’) and loss modulus (G”) of the reaction product of 10:1 mixture.

Viscoelasticity measurement of the reaction product was conducted under the conditions that temperature and strain were held constant. Figure 2 shows the data of storage modulus G’ and loss modulus G” as a function of angular frequency for a 10:1 mixture after the condensation reaction. G’ and G” increased with increasing angular frequency, and there was a crossover point at 100 rad/s. These graphs indicate that the resultant polymer shows a characteristic of viscous liquid below the crossover point frequency, and elastic contribution dominates over the frequency [16].

### 3.2. Microencapsulation

**Figure 3:**
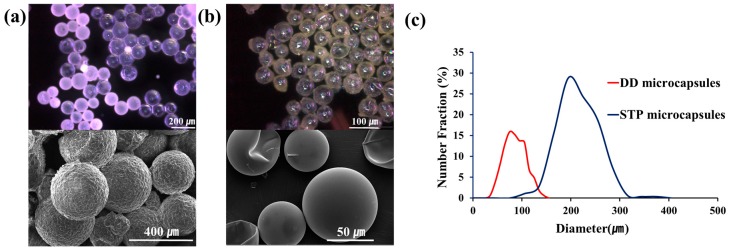
(**a**) Optical microscope (**top**) and SEM images (**bottom**) of the STP-containing microcapsules; (**b**) optical microscope (**top**) and SEM images (**bottom**) of the DD-containing microcapsules; (**c**) microcapsule size distribution of STP and DD microcapsules prepared at 1000 rpm.

Microencapsulation of STP and DD was conducted using urea-formaldehyde polymer (UF) and polyurethane (PU) as shell materials, respectively. The PU microcapsules containing DD were prepared by interfacial polymerization, and the UF microcapsules containing STP by in-situ polymerization. The morphology of the microcapsules was investigated by optical microscopy and scanning electron microscopy (SEM) (Figure 3a,b). Spherical microcapsules were obtained at an agitation rate of 1000 rpm. The outer surface of the UF capsules was relatively rough, and that of the PU capsules was smooth. The average capsule diameters of DD- and STP-loaded microcapsule were 75 and 220 μm, respectively (Figure 3c). Because the 8:1–12:1 mass ratio of STP:DD is required, the size of DD-loaded microcapsules is desired to be much smaller than that of STP microcapsules. If the size of microcapsules is smaller, more microcapsules can be contained in a coating, resulting in an increased probability of microcapsule rupture upon damaging.

The formation of STP- and DD-loaded microcapsules was confirmed by FT-IR spectroscopy (Figures S2 and S3 in Supplementary material). The IR spectrum of an authentic STP is practically the same as that of a substance that was released from the UF microcapsules when they were crushed by pressing with a spatula. This result indicates the successful formation of STP-loaded UF microcapsules. The formation of DD-loaded PU microcapsules was also confirmed in a similar fashion (Figure S3). It was found that the STP-loaded microcapsules have a core/shell mass ratio of 66:34. The DD-loaded microcapsules have a chlorobenzene/DD/shell mass ratio of 66:16:18, which indicates that a large portion of chlorobenzene evaporated during the microencapsulation.

### 3.3. SEM Study

**Figure 4:**
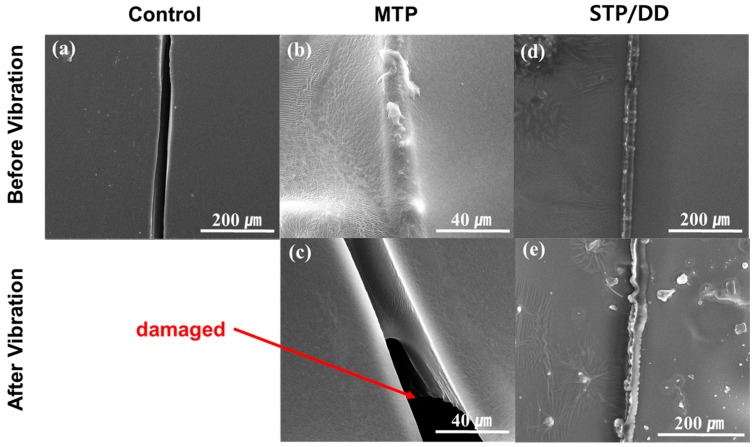
SEM images of the scratched region of (**a**) a control coating; (**b**) methacryloxypropyl-terminated polydimethylsiloxane (MTP)-based self-healing coating after scratching and healing; (**c**) MTP-based self-healing coating after scratching, healing, and vibration; (**d**) STP/DD-based self-healing coating after scratching and healing; and (**e**) STP/DD-based self-healing coating after scratching, healing and vibration.

The STP- and DD-containing microcapsules were mixed into a commercial enamel paint formulation with a mass ratio of STP capsules:DD capsules:paint of 19:6:75 to prepare self-healing coatings. The 19:6 ratio of STP capsules:DD capsules corresponds to a 12:1 mass ratio of STP:DD. Control coating samples were also prepared without microcapsules. For comparison, methacryloxypropyl-terminated polydimethylsiloxane (MTP) was employed as a healing agent, which generates a hard solid through photo-cross-linking [10]. MTP was microencapsulated to prepare comparison self-healing coatings. Each coating formulation was applied to one side of a silicon wafer to make a sample for SEM study. The STP/DD-based self-healing coatings were scratched with a razor blade and left for 4 h to induce the reaction of STP. In the case of scratched MTP-based coatings, photoirradiation was conducted with a xenon lamp. From the SEM images of the scratched areas in the STP/DD- and MTP-based self-healing coatings, it was confirmed that the healing agents filled the scratched region, indicating effective healing of the scratch (Figure 4d,b, respectively), while the scratch remained unfilled in the case of the control coating without microcapsules (Figure 4a). Upon the application of vibration of 10 to 30 Hz for 1 h, secondary damage occurred in the healed region of the MTP-based coating, and some fragments of the solidified healing agent fell out (Figure 4c). In contrast, no damage was observed in the STP/DD-based coating upon vibration (Figure 4e). This is considered to be due to the viscoelasticity of the substance generated from the STP/DD healing agent. On the other hand, in the unscratched regions of the coatings, no damage occurred upon the vibration application.

### 3.4. Electrochemical Test

**Figure 5:**
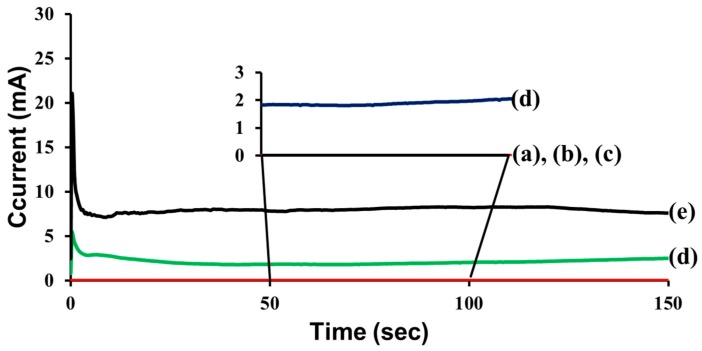
Current versus time for scratched self-healing and control coatings: (**a**) STP/DD-based coating before vibration; (**b**) MTP-based coating before vibration; (**c**) STP/DD-based coating after vibration; (**d**) MTP-based coating after vibration; and (**e**) control coating.

Electrochemical testing provides further evidence of the self-healing and the secondary damage-preventing function of the self-healing coatings (Figure 5). The STP and DD-loaded microcapsules were dispersed in a commercial epoxy coating formulation, and the resultant mixture was applied to steel panels. The self-healing coatings were damaged by scratching with a razor blade; it was confirmed that each cut was deep enough to reach the surface of the steel panel. The electrochemical test was conducted in a conventional three-electrode electrochemical cell; the coated steel panel served as the working electrode (Figure S1 in Supplementary Material). The steady-state conduction between the coated steel substrate and a counter electrode held at 3 V through an aqueous electrolyte (1 M NaCl) was measured. The scratched control coating showed a current flow of 8.0 mA (Figure 5e), indicating that the damage was not healed. The scratched STP/DD and MTP-based self-healing coating samples exhibited very low current values of 3.7 and 5.2 nA, respectively (Figure 5a,b). The results indicate that self-healing occurred in those STP/DD- and MTP-based coatings. After applying vibration to the scratched and healed self-healing coatings, the current flow of MTP-based coating increased to 2.2 mA after vibration (Figure 5d), while the current value of STP/DD-based coating remained near zero (5.2 nA) (Figure 5c). These results show that MTP-based coating samples were damaged by the vibration, and the secondary damage could not self-heal. In contrast, vibration-induced secondary damage could evidently be prevented in the STP/DD-based coating. The electrochemical test results are in accord with the SEM study results described above. Repeated electrochemical tests showed good reproducibility.

### 3.5. Water Permeability Test

**Figure 6:**
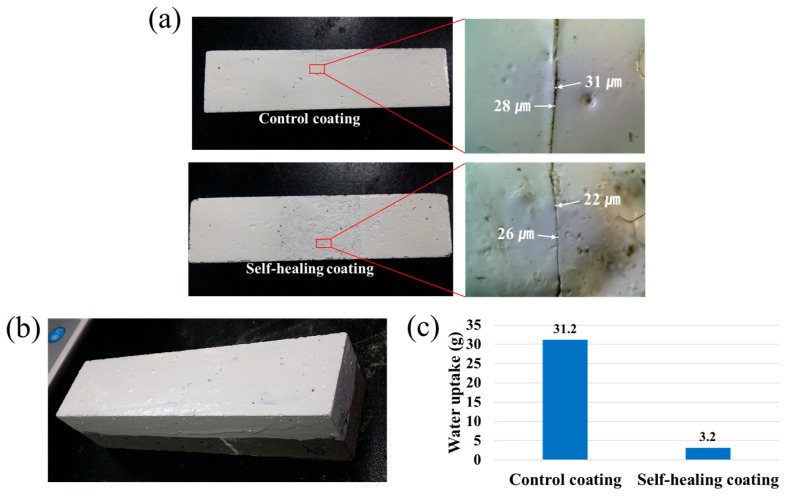
Water permeability test for coated mortar specimens. (**a**) Photographs of scratched coatings (**left**) and enlarged scratch areas (**right**); scratch widths are indicated; (**b**) A photograph of coated mortar specimen; (**c**) Amount of water uptake upon immersion of the scratched surface in water for 48 h.

In order to preliminarily evaluate the applicability of the STP/DD-based self-healing coating system to cementitious composite material, a water permeability test was conducted using mortar specimens (Figure 6). Each specimen was prepared by the application of the self-healing coating to one rectangular side of a square column mortar (Figure 6b), and by scratching the coated surface of the mortar with a razor blade (Figure 6a). It was confirmed that each cut was deep enough to reach the mortar surface. Optical microscopy shows that the scratch width ranges from 20 to 35 μm. The scratched self-healing coating was allowed to heal for 4 h. For comparison, control coating samples were prepared in a similar fashion. Figure 6c shows that the amount of water uptake of the scratched control specimen was 31.2 g. When the self-healing coating was scratched, the water uptake amount corresponded to only 10% of the water uptake of the scratched control coating sample. The results suggest that successful self-healing of damage was accomplished in the self-healing system, leading to the effective prevention of water permeation.

## 4. Conclusions

Viscoelastic materials were generated from STP/DD mixtures with a mass ratio of STP:DD = 8:1–12:1. STP and DD were separately microencapsulated, and the mixed microcapsules were integrated into enamel paint or epoxy coating formulations to prepare a dual-capsule self-healing coating. The self-healing function of the coating was demonstrated by SEM and electrochemical test. It is considered that when the self-healing coating is scratched, STP and DD are released from ruptured microcapsules, fill the scratched region, and turn into a viscoelastic material. It was confirmed that secondary crack does not occur in the healed region upon the application of vigorous vibration to the self-healing coating. The applicability of the self-healing coating system to mortar specimen was successfully demonstrated by water permeability test.

## Supplementary Materials

The following are available online at www.mdpi.com/1996-1944/10/2/114/s1. Figure S1: Schematic diagram of electrochemical test. Figure S2: Infrared spectra of (a) an authentic STP and (b) the core that flowed out of ruptured UF microcapsules. Figure S3: Infrared spectra of (a) an authentic DD and (b) the core that flowed out of ruptured PU microcapsules. Figure S4: Procedure for water permeability test: (a) scratching a coating on a mortar specimen; (b) weighing; (c) immersion of coated side of the specimen in water; (d) taking the specimen out of a water bath; (e) wiping the immersed surfaces and drying; and (f) weighing.
