# Supplementary material for: Microcapsule-Type Self-Healing Protective Coating for Cementitious Composites with Secondary Crack Preventing Ability
Source: Materials (Basel). 2017 Jan 26;10(2):114. doi: 10.3390/ma10020114 (PMC5459122; doi:10.3390/ma10020114)
Supplement: Supplementary file 1 [file materials-10-00114-s001.pdf]

Supplementary Materials: Microcapsule-Type Self-Healing Protective Coating for Cementitious Composites with Secondary Crack Preventing Ability

Dong-Min Kim, Hwan-Chul Yu, Hye-In Yang, Yu-Jin Cho, Kwang-Myong Lee and Chan-Moon Chung

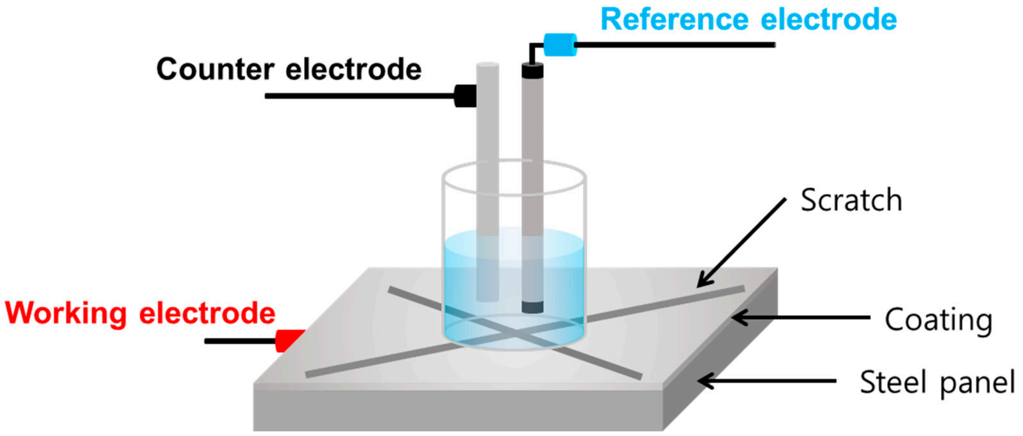

Figure S1. Schematic diagram of electrochemical test.

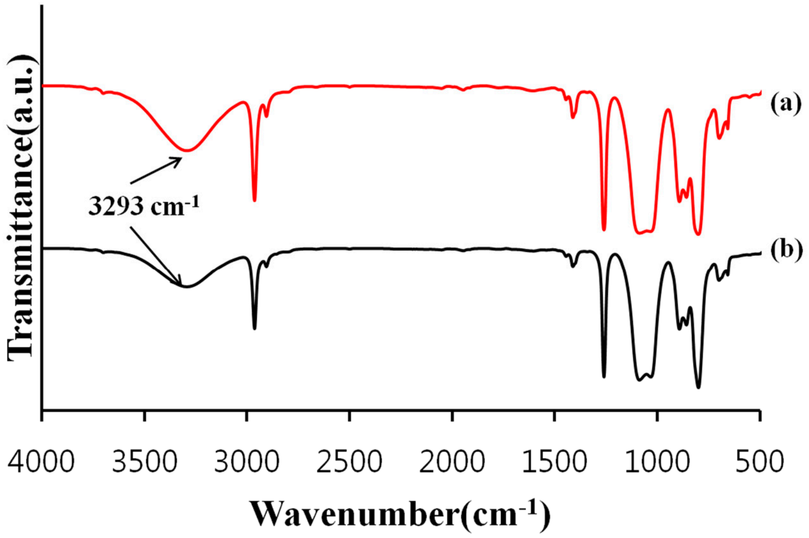

Figure S2. Infrared spectra of (**a**) an authentic silanol-terminated polydimethylsiloxane (STP) and (**b**) the core that flowed out of ruptured urea-formaldehyde (UF) microcapsules.

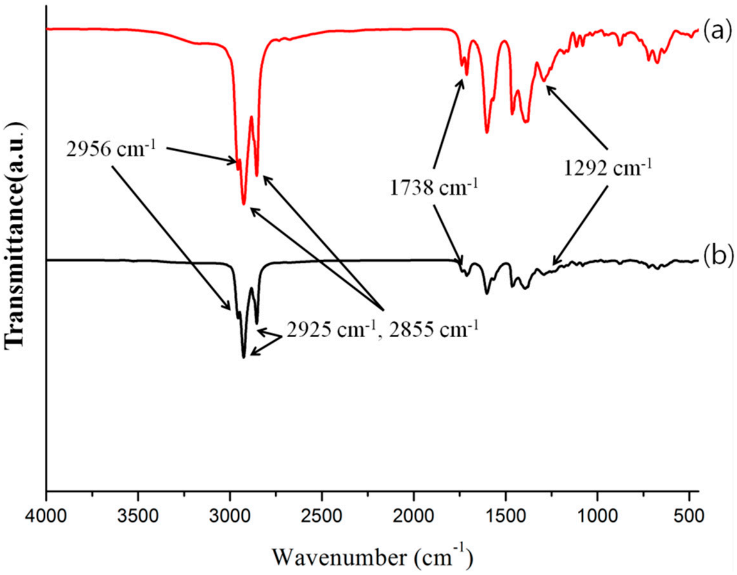

Figure S3. Infrared spectra of (**a**) an authentic dibutyltin dilaurate (DD) and (**b**) the core that flowed out of ruptured polyurethane (PU) microcapsules.

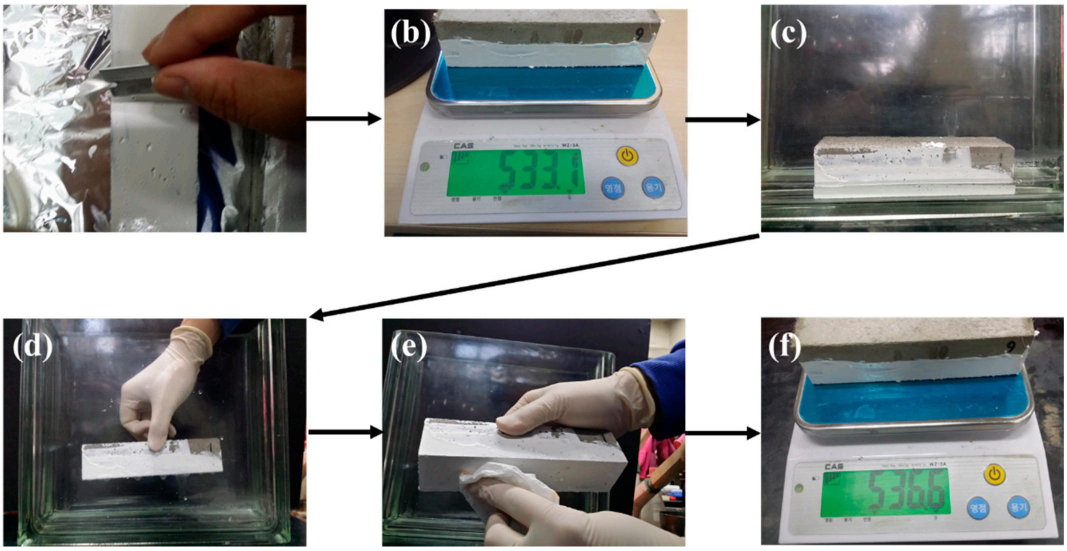

Figure S4. Procedure for water permeability test: (**a**) scratching a coating on a mortar specimen; (**b**) weighing; (**c**) immersion of coated side of the specimen in water; (**d**) taking the specimen out of a water bath; (**e**) wiping the immersed surfaces and drying; and (**f**) weighing.
